# Acute acalculous cholecystitis and blackwater fever with acute kidney injury in children with malaria: case series and literature review

**DOI:** 10.1186/s12936-026-06013-9

**Published:** 2026-06-18

**Authors:** Marco Cugliari, Francesca Piacentini, Sara M. M. A. Montorfani, Obalim Fiddy, Sara Testa

**Affiliations:** 1 Department of Clinical Science and Community Health, University of Milan, Milan, Italy; 2 Pediatric Nephrology, Dialysis and Transplantation Unit, Fondazione IRCCS Ca’ Granda Ospedale Maggiore Policlinico, Milan, Italy; 3 Department of Health Science, University of Florence, Florence, Italy; 4 Faculty of Medicine, University of Fribourg, Fribourg, Switzerland; 5 Paediatric department, St. Mary’s Hospital Lacor, Gulu, Uganda

**Keywords:** Malaria, Acute acalculous cholecystitis, Blackwater fever, Acute kidney injury, Pediatrics, Infectious diseases, Tropical medicine, Case series, Case report, East Africa

## Abstract

**Background:**

Acute acalculous cholecystitis (AAC) is an inflammatory gallbladder disease occurring in the absence of gallstones. It typically presents with right upper quadrant (RUQ) pain, fever and gastrointestinal symptoms, and is diagnosed by ultrasound evidence of cholecystitis without gallstones. AAC can have multiple causes, including infections, with malaria only rarely reported.

**Case presentations:**

We describe four Ugandan children who developed malaria-associated AAC. They were all referred from other centers and presented at a more advanced stage of disease. Apart from AAC, they also presented blackwater fever (BWF) and severe acute kidney injury (AKI), complications increasingly recognized in malaria. AAC was managed conservatively, while two patients required dialysis for kidney failure. All patients ultimately recovered both gallbladder and renal function.

**Conclusions:**

We provide a review of the literature on malaria-associated AAC, which identified 28 previously reported cases, only 7 in children. Most reported patients contracted the infection in malaria-endemic regions but were then managed in high-income countries. The majority were managed conservatively and had favorable outcomes. Including our series, 32 cases (11 pediatric) have now been reported. Our findings suggest that AAC, which can often occur in association with BWF and AKI, is likely underrecognized in malaria-endemic regions. Heightened clinical awareness and early assessment are important, and practical management recommendations are provided.

## Introduction

Acute acalculous cholecystitis (AAC) is an inflammatory disease of the gallbladder characterized by absence of gallstones. While it accounts for only 5–10% of cases of cholecystitis in adults, it represents 50–70% of cases of cholecystitis in childhood [1]. The main underlying mechanisms are bile stasis (following prolonged fasting, parenteral nutrition or severe dehydration) and local ischemia, leading to inflammation and tissue necrosis. These conditions are more common in critically ill patients, for example following major surgery (more commonly described in adults), extensive burns and shock syndromes. Cases of pediatric AAC have also been associated with immune-mediated disorders and infections, mainly viral, whereas malaria has only rarely been implicated. Management is not standardized but is usually conservative, especially in pediatric patients, with generally favorable outcomes [1–3].

Many of the factors implicated in AAC pathogenesis are common in malaria, such as systemic inflammation, fasting and dehydration, often leading to volume depletion and in some cases shock, microvascular ischemia. Sequestration of parasites in the gallbladder microvasculature is also believed to play a role [4–6].

BWF is a complication of malaria, characterized by intravascular hemolysis, fever, and passage of dark-colored urine. Although AAC and BWF are considered rare complications of malaria, we report four pediatric cases, all presenting with AAC, BWF and AKI, observed within a single week in Gulu, northern Uganda, a region with a high malaria burden (up to 90 cases/1000 individuals at risk per month, in the highest burden months of the year) [7].

To our knowledge, concurrent occurrence of AAC, BWF, and AKI in multiple pediatric malaria cases within a short timeframe has not been previously reported. This case series aims to describe these cases and contextualize them within existing literature.

## Case presentations

Cases are summarized in Table 1

**Table 1:** Characteristics of our four patients

	Age (years)	Gender	Plasmodium species	Symptoms	Key lab findings at presentation	Ultrasound results	AAC developed before or after start of antimalarial treatment	BWF	AKI	Treatment	Outcome
Patient 1	12	Female	*P*. *falciparum*	Fever, abdominal pain, RUQ tenderness, dark-coloured urine	Hb 8.5 g/dl; WBC 7.7 × 10^9^/L; creatinine 17.5 mg/dl; total bilirubin 1.5 mg/dl	Thickened gallbladder wall (4 mm), positive sonographic Murphy sign	After	Yes	Yes	Antibiotics	Recovery of AAC, transferred to another hospital to start peritoneal dialysis
Patient 2	7	Female	*P*. *falciparum*	Abdominal pain, RUQ tenderness, jaundice, dark-colored urine, oliguria	Hb 7.8 g/dl; WBC 9 × 10^9^/L; creatinine 9.1 mg/dl; total bilirubin 28 mg/dl; ALT 339 U/L; AST 481 U/L	Thickened gallbladder wall (10.4 mm), distended gallbladder, sonographic Murphy sign	After	Yes	Yes	Antibiotics, UDCA, peritoneal dialysis	Complete recovery
Patient 3	9	Female	*P*. *falciparum*	Fever, abdominal pain, dark-colored urine, oliguria, generalized edema, lethargy	Hb 7.4 g/dl; WBC 8.2 × 10^9^/L; creatinine 16.5 mg/dl; total bilirubin 2.7 mg/dl	Thickened gallbladder wall (12.8 mm), pericholecystic fluid collection, sonographic Murphy sign	After	Yes	Yes	Antibiotics	Complete recovery
Patient 4	10	Male	*P*. *falciparum*	Fever, abdominal pain, RUQ tenderness, vomiting, diarrhea, dark-colored urine	Hb 3.3 g/dl; WBC 4.5 × 10^9^/L; creatinine 21.4 mg/dl; total bilirubin 2.1 mg/dl	Thickened gallbladder wall, distended gallbladder, sonographic Murphy sign	After	Yes	Yes	Antibiotics	Complete recovery

AAC, acute acalculous cholecystitis; AKI, acute kidney injury; ALT, alanine aminotransferase; AST, aspartate aminotransferase; BWF, blackwater fever; RUQ, right upper quadrant; UDCA, ursodeoxycholic acid

### Case 1

A 12-year-old female patient was transferred to our hospital from a health center with a two-week history of fever, abdominal pain, dark-colored urine, generalized edema. Blood smear microscopy was positive for malaria and she was treated before referral with a full cycle of antimalarials and blood transfusion. Upon presentation, abdominal ultrasound revealed enlarged kidneys with increased echogenicity and enlarged gallbladder with thickened walls (4 mm) in the absence of gallstones, sonographic Murphy sign was positive. Blood exams showed markedly increased renal function tests (RFTs, serum creatinine 17.5 mg/dl). Malaria rapid diagnostic test (MRDT) was positive, while blood smear microscopy was negative. Blood culture later turned out to be negative. She was treated with analgesics, antibiotic therapy (ceftriaxone plus metronidazole), and medical management of renal failure. She became afebrile the day after starting antibiotic therapy, while abdominal pain resolved after four days. Despite medical treatment, renal failure persisted, so the patient was transferred to another hospital to start peritoneal dialysis.

### Case 2

A 7-year-old female was transferred for further management of AKI from another center, where she tested positive for malaria MRDT and received IV artesunate followed by oral artemether-lumefantrine. Upon arrival at our center, she had jaundice, peripheral edema, abdominal pain with RUQ tenderness and oliguria with dark-colored urine. Blood exams showed increased RFTs (creatinine 9.1 mg/dl) and liver function tests (LFTs), with markedly increased bilirubin (ALT 339 U/L, AST 481 U/L, total bilirubin 28.1 mg/dl, with direct bilirubin 22.6 mg/dl). Blood smear microscopy was negative for malaria. Abdominal ultrasound showed hyperechogenic kidneys, pleural effusion, ascites, and a distended gallbladder with a thickened wall of 10.4 mm and positive sonographic Murphy sign. She was treated with analgesics, ursodeoxycholic acid, antibiotics (ceftriaxone and metronidazole) and medical management of renal failure. Abdominal pain subsided on day 5 after admission. On day 6, she was started on peritoneal dialysis for renal failure. LFTs normalized on day 7. Her kidney function gradually improved, and dialysis was stopped on day 12. She was then discharged home.

### Case 3

A 9-year-old female was transferred from another center for management of AKI. She had developed oliguria with dark-colored urine after being managed for malaria (diagnosed by MRDT test). Upon arrival at our hospital, she presented with fever, oliguria, generalized edema with ascites, and lethargy. Blood exams showed increased RFTs (creatinine 16.5 mg/dl), mild elevation of bilirubin (2.7 mg/dl total) and gamma-glutamyl transferase (177 U/L), other LFTs were normal. Blood smear microscopy was negative for malaria. Blood culture later turned out to be negative. Ultrasound showed increased renal echogenicity with loss of corticomedullary differentiation, ascites, a distended gallbladder with a thickened wall (12.8 mm), pericholecystic fluid collection and positive sonographic Murphy sign. She received analgesics, ceftriaxone, metronidazole and medical management for AKI. After an initial improvement, she developed pneumonia on day 7 after admission. Linezolid was added, with resolution of fever after 3 days. She was then discharged home.

### Case 4

A 10-year-old male presented with fever, anemia, abdominal pain, vomiting, diarrhea and dark-colored urine. MRDT and blood smear microscopy were positive for malaria. The patient was given IV artesunate, followed by piperaquine/dihydroartemisinin and blood transfusion. After a full cycle of antimalarials the patient was still febrile and weak, urine output was reduced and abdominal pain worsened, with positive Murphy sign. Blood exams showed increased white blood cell count (WBC 24.2 × 10.^9^/L, neutrophils 13.5 × 10^9^/L), increased RFTs (creatinine 14.2 mg/dL) and LFTs (ALT 137 U/L, AST 700 U/L), with only mild elevation of bilirubin (2.1 mg/dl total). Ultrasound showed increased renal echogenicity with decreased corticomedullary differentiation, mild pericardial effusion, enlarged gallbladder with thickened walls and positive sonographic Murphy sign. Blood culture later turned out to be negative. Ceftriaxone and metronidazole were started. After 3 days there was still little improvement, so Meropenem was started, with resolution of fever and abdominal pain on day 5. LFTs normalized by day 8. The patient gradually recovered from AKI in the following weeks and was discharged home on day 21.

## Discussion

We described four pediatric patients with malaria who developed AAC, a complication considered to be rare. AAC typically presents with fever, RUQ pain with positive Murphy sign, and gastrointestinal symptoms such as nausea and vomiting. Jaundice is rare but can be present, mainly due to sepsis-related cholestasis or inflammation leading to biliary obstruction. Blood exams can show increased white blood cell count and inflammatory markers, elevated LFTs and bilirubin [1, 2]. Diagnosis is confirmed by ultrasound, with typical findings being absence of stones in the gallbladder, positive sonographic Murphy sign, increased gallbladder wall thickness (> 3.5 mm), gallbladder enlargement, and pericholecystic fluid collection. Bile sludge can also be present. Thickening of the gallbladder wall is the most reliable criterion [1–3]. Unlike adults, AAC in children is mainly managed conservatively, with treatments typically including antibiotics, analgesics, fluid and electrolyte support, and fasting [1, 2]. UDCA has also been used in a few cases of AAC, with proposed benefits being anti-inflammatory effect, reduction of biliary sludge and biliary pain. Antibiotic coverage is justified by the possibility of bacterial infections causing AAC, along with the common presence of high fever and leukocytosis. In cases of AAC managed with biliary drainage, bacterial isolates have been described. Therapy should be directed against Gram negative bacteria and anaerobes. Unlike other causes of AAC, malaria-associated AAC is only rarely managed surgically [1–3, 8].

Interestingly, all our patients presented BWF and AKI in addition to AAC. BWF was historically described in nonimmune expatriates from Europe going to tropical regions and getting infected with *Plasmodium falciparum* [9]. One of the first reported risk factors for BWF was prior treatment with quinine, an antimalarial associated with a high rate of hemolysis. Another associated risk factor is G6PD deficiency [9, 10].The incidence of this disease markedly decreased during the twentieth century, but recent reports highlight an increasing incidence of BWF, especially in autochthonous children from East Africa [10–13]. BWF has been associated with an increased risk of severe AKI, severe anemia, poorer clinical conditions and worse post discharge outcomes. Mortality rates are often reported to be similar in children with and without BWF. However, only in-hospital mortality has been assessed in these studies, and long-term data are lacking. In a study with a 6-month follow-up, patients with severe anemia who presented BWF had a two times higher incidence of readmission and three times higher incidence of death compared to patients who had severe anemia without BWF [9, 11–13]. Development of BWF in children is more common in cases of recent malarial infection, previous blood transfusion, or recent antimalarial treatment [11, 13]. Children with BWF have an increased incidence of readmission, especially if they also have severe anemia. The main cause of readmission is a new malaria infection in the months following discharge. Based on these findings, it has been suggested that children with BWF receive post-discharge malaria prophylaxis for at least 6 months, especially those who also develop AKI and severe anemia [13]. There is no specific treatment described for BWF; cases are managed symptomatically according to local practices for severe malaria [13, 14]. Recent studies show that AKI is a common complication of severe malaria, with estimated incidence of up to half of children hospitalized with severe malaria. Early recognition of patients with AKI or at high risk of AKI is fundamental to mitigate worsening of kidney function and disease progression. Management of malaria-AKI is similar to non-malarial AKI, including withdrawal of nephrotoxic medications, hemodynamic monitoring, monitoring of serum creatinine and urine output, ensuring adequate volume status, management of electrolyte abnormalities. Furthermore, in-hospital administration of paracetamol in patients with malaria-AKI has been associated with a reduced risk of acute kidney disease at 1-month follow-up. In case of failure of medical therapy, with refractory fluid overload, electrolyte abnormalities or uremic complications, renal replacement therapy with dialysis is recommended, and should be started as early as possible when indicated [15–18].

We performed a review of the literature regarding AAC associated with malaria. PubMed, Embase, Scopus and Web of Science were searched using the terms”acute acalculous cholecystitis” or “acalculous cholecystitis” combined with “malaria” or “plasmodium”. We identified 20 published articles, describing a total of 28 cases of AAC associated with malaria (Table 2) [4–6, 19–35]. Out of these cases, 7 were pediatric patients [6, 31–35]. Including our four cases, 32 total patients (11 pediatric) have been reported. 50% of patients were females, and the median age was 26 years (IQR 9–45). In line with what is known in the literature, the main features of AAC in the reported cases were RUQ tenderness and increased gallbladder thickness on ultrasound. Blackwater fever had only been reported in one case before our four patients. Interestingly, at least half of the reported cases had also AKI, highlighting a strong association between AAC and AKI in malaria. AAC occurred in similar frequency before and after initiation of antimalarial therapy, without a clear temporal pattern suggesting that treatment itself was the main trigger. Most cases were managed conservatively, with only four patients receiving surgical treatment. Patients presented with varying degrees of severity; however, AAC resolved without sequelae in all cases, underscoring the favorable prognosis of this condition.

**Table 2:** Characteristics of the 28 reported cases of acute acalculous cholecystitis associated with malaria

First author [Ref]	Age in years	Gender	Country (where infection was contracted)	Autochthonous/Traveller	Malaria prophylaxis	Symptoms	Ultrasound results	AAC developed before or after start of antimalarial treatment	BWF	AKI	Treatment	Outcome
Dylewski 1999 [19]	26	Female	Togo	Traveller	No	Abdominal pain, nausea, vomiting, fever, hypotension, lethargy	Thickened gallbladder wall (5 mm), pericholecystic fluid collection	Before	No	Unknown	IV fluids, antibiotics, antimalarials	Complete recovery
Sanchez 2000 [20]	24	Female	Dominican Republic	Traveller	No	Fever, diarrhea, hypotension, lethargy, right upper quadrant tenderness	Thickened gallbladder wall, pericholecystic fluid collection	Before	No	No	Antibiotics, antimalarials	Complete recovery
Maggi 2002 [21]	46	Female	Nigeria	Traveller	No	Abdominal pain, vomiting, diarrhea, fever, lethargy, RUQ tnderness	Thickened gallbladder wall (5 mm), pericholecystic fluid collection, bile sludge	Before	No	Yes	Antibiotics, antimalarials	Complete recovery
Saha 2005 [31] *	7	Female	India	Autochthonous	No	Fever, headache, RUQ tenderness	Thickened gallbladder wall, pericholecystic fluid collection	Before	No	No	Antibiotics, antimalarials, blood transfusion	Complete recovery
Kuttiat 2006 (case 1) [32] *	8	Male	India	Autochthonous	No	Fever, RUQ tenderness, hypotension, lethargy	Thickened gallbladder wall, pericholecystic fluid collection, bile sludge	Before	No	No	IV fluids, antibiotics, antimalarials, blood transfusion	Complete recovery
Kuttiat 2006 (case 2) [32] *	9	Male	India	Autochthonous	No	Fever, abdominal pain, RUQ tenderness, vomiting, hypotension, lethargy	Thickened gallbladder wall, pericholecystic fluid collection	Before	No	Unknown	IV fluids, antimalarials	Complete recovery
Yombi 2006 [22]	24	Female	Cameroon	Traveller	Unknown	Fever, abdominal pain, RUQ tenderness, vomiting, hypotension, headache	Thickened gallbladder wall (10 mm), sonographic Murphy sign	After	No	Unknown	Antibiotics, antimalarials	Complete recovery
Anthoine-Milhomme 2007 [33] *	7	Female	Ivory Coast	Traveller	No	Fever, abdominal pain, RUQ tenderness, diarrhea, jaundice, headache	Thickened gallbladder wall	After	Unknown	Unknown	IV fluids, antimalarials	Complete recovery
Kumar 2008 [34] *	3	Female	India	Autochthonous	No	Fever, abdominal pain, RUQ tenderness, vomiting	Thickened gallbladder wall (6 mm)	Before	No	No	Antibiotics, antimalarials, blood transfusion	Complete recovery
Sokwala 2008 [23]	64	Male	Uganda	Autochthonous	Unknown	Fever, abdominal pain, RUQ tenderness, vomiting, headache	Thickened gallbladder wall (7 mm), sonographic Murphy sound	After	No	Unknown	Antibiotics, antimalarials	Complete recovery
Khan 2009 [24]	40	Male	Eritrea	Autochthonous	No	Fever, abdominal pain, RUQ tenderness, vomiting	Thickened gallbladder wall (6 mm), pericholecystic fluid collection	Before	No	No	Antimalarials	Complete recovery
Salinas 2009 [25]	26	Female	Dominican Republic	Traveller	No	Fever, abdominal pain, RUQ tenderness, vomiting	Thickened gallbladder wall, pericholecystic fluid collection	After	No	Unknown	Antimalarials	Complete recovery
Carvalho 2011 [26]	53	Male	Malawi	Traveller	Yes	Fever, jaundice, hypotension	Thickened gallbladder wall, gallbladder distension, sonographic Murphy sign	After	No	Yes	IV fluids, antibiotics, antimalarials, percutaneous cholecystostomy	Worsening of clinical conditions with MOF, followed by gradual recovery
Curley 2011 (case 1) [5]	26	Male	Korea	Traveller	No	Fever, abdominal pain, diffuse abdominal tenderness, vomiting, hypotension, headache	Thickened gallbladder wall (4 mm)	Before	No	Unknown	IV fluids, antibiotics, antimalarials	Complete recovery
Curley 2011 (case 2) [5]	21	Male	Iraq	Traveller	Yes	Fever, lethargy	Thickened gallbladder wall (4.5 mm)	After	No	Unknown	IV fluids, antibiotics, antimalarials, percutaneous cholecystostomy	Worsening of clinical conditions with ARDS requiring intubation, followed by gradual recovery
Abreu 2013 (case 1) [4]	27–53 (median 45)	5 males, 2 females	Sub-Saharan Africa	Traveller	Unknown	Unspecified	Thickened gallbladder wall, pericholecystic fluid collection, sonographic Murphy sign	Unknown	Unknown	Yes	Antibiotics, antimalarials, two patients also received percutaneous cholecystostomy	Complete recovery
Abreu 2013 (case 2) [4]	27–53 (median 45)	5 males, 2 females	Sub-Saharan Africa	Traveller	Unknown	Unspecified	Thickened gallbladder wall, gallbladder wall edema	Unknown	Unknown	Yes	Antibiotics, antimalarials, two patients also received percutaneous cholecystostomy	Complete recovery
Abreu 2013 (case 3) [4]	27–53 (median 45)	5 males, 2 females	Sub-Saharan Africa	Traveller	Unknown	Unspecified	Thickened gallbladder wall, gallbladder wall edema	Unknown	Unknown	Yes	Antibiotics, antimalarials, two patients also received percutaneous cholecystostomy	Complete recovery
Abreu 2013 (case 4) [4]	27–53 (median 45)	5 males, 2 females	Sub-Saharan Africa	Traveller	Unknown	Unspecified	Thickened gallbladder wall, gallbladder wall edema, sonographic Murphy sign, bile sludge	Unknown	Unknown	Yes	Antibiotics, antimalarials, two patients also received percutaneous cholecystostomy	Complete recovery
Abreu 2013 (case 5) [4]	27–53 (median 45)	5 males, 2 females	Sub-Saharan Africa	Traveller	Unknown	Unspecified	Thickened gallbladder wall, intramural gas, bile sludge	Unknown	Unknown	Yes	Antibiotics, antimalarials, two patients also received percutaneous cholecystostomy	Complete recovery
Abreu 2013 (case 6) [4]	27–53 (median 45)	5 males, 2 females	Sub-Saharan Africa	Traveller	Unknown	Unspecified	Thickened gallbladder wall, pericholecystic fluid collection, bile sludge	Unknown	Unknown	Yes	Antibiotics, antimalarials, two patients also received percutaneous cholecystostomy	Complete recovery
Abreu 2013 (case 7) [4]	27–53 (median 45)	5 males, 2 females	Sub-Saharan Africa	Traveller	Unknown	Unspecified	Thickened gallbladder wall, gallbladder wall edema	Unknown	Unknown	Yes	Antibiotics, antimalarials, two patients also received percutaneous cholecystostomy	Complete recovery
Harris 2013 [27]	59	Male	Ghana	Traveller	No	Fever, abdominal pain, RUQ tenderness, vomiting	Thickened gallbladder wall, pericholecystic fluid collection	After	No	Unknown	Antibiotics, antimalarials	Complete recovery
Colomba 2017 [28]	52	Female	Ghana	Autochthonous	Unknown	Fever, abdominal pain, RUQ tenderness	Thickened gallbladder wall (3.5 mm), pericholecystic fluid collection	Before	No	Unknown	Antibiotics, antimalarials	Complete recovery
Aguilera-Alonso 2018 [6] *	5	Female	Equatorial Guinea	Autochthonous	No	Fever, RUQ tenderness, jaundice	Thickened gallbladder wall (4.5 mm), bile sludge	After	Unknown	Yes	IV fluids, antibiotics, antimalarials	Complete recovery
El Khader 2019 [29]	58	Male	Ivory Coast	Traveller	Unknown	Fever, abdominal pain, RUQ tenderness, vomiting, diarrhea, jaundice	Thickened gallbladder wall (5.4 mm)	Before	No	Yes	Antibiotics, antimalarials	Complete recovery
Greer 2020 [35] *	4	Female	Tanzania	Autochthonous	Unknown	Abdominal pain, RUQ tenderness, vomiting	Thickened gallbladder wall (6 mm), pericholecystic fluid collection, dilated common bile duct	Before	No	Unknown	Antimalarials	Complete recovery
Hanif 2023 [30]	24	Male	Sierra Leone	Autochthonous	Unknown	Fever, RUQ tenderness, lethargy, headache	Thickened gallbladder wall	After	Yes	Yes	Antimalarials, plasma exchange, hemodialysis	Recovery with persistent kidney impairment

^*^Pediatric cases

AAC, acute acalculous cholecystitis; AKI, acute kidney injury; BWF, blackwater fever; RUQ, right upper quadrant

Most of previously reported cases were isolated. Our series is the first one describing several cases of malaria-associated AAC occurring over a short time frame. This clustering may reflect the combined effect of delayed access to care, late referral from peripheral facilities, high local malaria transmission, and limited early access to ultrasound in most facilities, rather than a distinct local predisposition to AAC. Also, considering that most cases in the literature are imported cases, it’s possible that malaria-associated AAC is much more common in developing countries than previously thought, as demonstrated by our series, but remains underreported.

The concurrent development of AAC and BWF with AKI in our cases may reflect the fact that all three occur in severe malaria, especially when access to care is delayed. However, this finding could also be explained by shared underlying mechanisms, which include fasting, dehydration, systemic inflammation, severe hemolysis, and sequestration of parasites in the microvasculature with consequent microvascular ischemia. Moreover, the inflammation associated with the uremic state of renal failure could increase the risk of AAC. The potential role of antimalarial therapy in the development of AAC remains to be determined. Given the overlap of these complications in our patients, clinicians might consider early abdominal ultrasound in patients with severe malaria who present with right upper quadrant pain or unexplained abdominal tenderness, especially in the presence of AKI.

Limitations of our study include its small and retrospective nature from a single center, limiting the generalizability of our findings to other children with malaria. Second, some diagnostic information is incomplete because patients were referred from peripheral facilities. Finally, although the temporal association between AAC, BWF, AKI, and severe malaria is notable, this study cannot establish causality or define the underlying pathophysiological mechanisms.

## Conclusion

The findings we described support the growing evidence that BWF is a common complication among children infected with malaria in East Africa, with a high risk for AKI and worse prognosis. They also suggest that AAC may be more common than previously thought in this population. Limited awareness of these complications, diagnostic challenges, and underreporting may contribute to this perception. There is also less tendency to publish such cases from developing countries; indeed, most reports describe patients managed after arrival to high-income countries. Knowledge of these complications is important for clinicians managing malaria all over the world, particularly in endemic regions, possibly leading to targeted treatment and prevention strategies for a more fragile subset of patients with malaria. Particular attention regarding renal involvement in malaria is needed, with early monitoring and timely referral for dialysis. Post-discharge malaria prophylaxis should be considered for patients with malaria who develop BWF and AKI. AAC should be considered when managing malaria cases with suggestive symptoms such as RUQ pain. Further efforts are required to better define pathophysiological mechanisms, assess the incidence in endemic populations, and standardize management.

## Data Availability

Further information is available from the corresponding author on reasonable request as no confidential information will be disclosed.
